# A Case Report on Pulmonary Tumor Embolism in a Patient With a Metastatic Gastrointestinal Stromal Tumor Invading the Inferior Vena Cava: Tumor or Thrombus?

**DOI:** 10.7759/cureus.80684

**Published:** 2025-03-16

**Authors:** Toshali Pandey, Mazin Safar

**Affiliations:** 1 Internal Medicine, University of Arkansas for Medical Sciences, Little Rock, USA; 2 Hematology and Medical Oncology, University of Arkansas for Medical Sciences, Little Rock, USA

**Keywords:** gastrointestinal stromal tumors, pulmonary cavity, pulmonary embolism, pulmonary infarction, tumor embolism, tumor thrombus

## Abstract

Pulmonary tumor embolism is a highly underdiagnosed entity involving the embolism of tumor fragments into the pulmonary vasculature. The vast majority of cases are diagnosed post-mortem. Here we discuss a rare case of a patient with a long-standing history of metastatic gastrointestinal stromal tumor (GIST) that progressed into a direct invasion of the inferior vena cava. The patient presented with shortness of breath and chest pain and was found to have a pulmonary embolism while on anticoagulation. Clinical and imaging findings strongly suggested tumor embolism. Due to its similarity to venous thromboembolism, pulmonary tumor embolism is challenging to diagnose antemortem. Clues to the diagnosis include the presence of intravascular invasion by the tumor (tumor thrombus), recurrent embolisms on anticoagulation, chronic vascular occlusion, and pulmonary infarction.

## Introduction

Pulmonary tumor embolism (PTE) is a rarely diagnosed entity involving the embolism of tumor fragments that have dislodged from the primary tumor into the pulmonary vasculature. These fragments are not contiguous with metastatic foci [1]. PTE exists across a spectrum ranging from macroembolism that mimics acute pulmonary thromboembolism to pulmonary tumor thrombotic microangiopathy (PTTM) [1,2]. PTTM is the result of chronic pulmonary microvascular occlusion due to the tumor embolism occurring in small arteries and arterioles with concomitant fibrocellular intimal proliferation.

Antemortem diagnosis of PTE is highly challenging due to the clinical and imaging overlap with venous thromboembolism (VTE). A majority of diagnoses are made either following autopsy or in rare instances, following mechanical thrombectomy in the antemortem setting [3].

Here we present a case of a patient with metastatic gastrointestinal stromal tumor (GIST) who presented with what appeared to be prima facie pulmonary thromboembolism. We also discuss the clinical and imaging characteristics that can point toward a diagnosis of tumor embolism instead.

## Case presentation

An 80-year-old man with a metastatic GIST, chronic obstructive pulmonary disease with chronic respiratory failure, and recurrent pulmonary embolisms on anticoagulation presented to the emergency department with shortness of breath and chest pain from the last 24 hours. 

**Figure 1:**
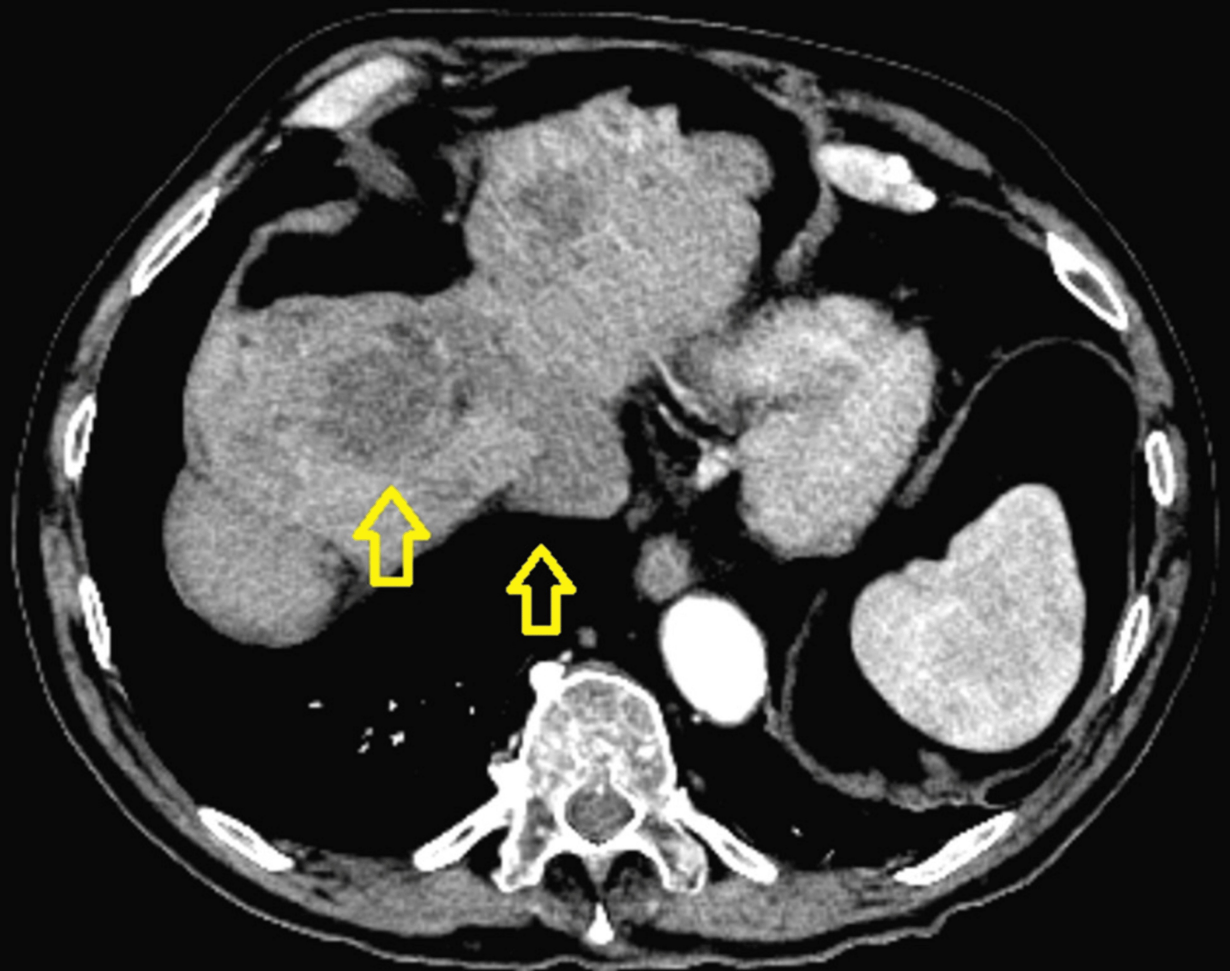
CT of the abdomen shows the liver with evidence of resection of segment four Multiple, scattered hypoattenuating lesions were demonstrated across the liver of which two are visible in this cross-section. The largest lesion measured 5.5 cm x 4.3 cm in the antero-posterior by transverse dimensions, respectively. A tumor thrombus is clearly visible emerging from a liver lesion distending the middle hepatic vein to an extent of 2.5 cm transversely and extending into the inferior vena cava (arrows).

He was diagnosed with GIST of his small bowel about 26 years ago. This was surgically resected. The tumor recurred in the form of a large solitary hepatic metastatic deposit about nine years later. This was proven by biopsy to be GIST. His tumor failed to respond to a trial of imatinib and was surgically resected. Four years later, the tumor recurred in the form of multiple hepatic metastatic lesions that were not amenable to resection. He was started on sunitinib and maintained stable disease on serial scans till a few months before presentation when a computed tomography (CT) scan of his abdomen revealed an increase in the size of his lesions and intravascular invasion of the inferior vena cava (IVC) (Figure 1).

**Figure 2:**
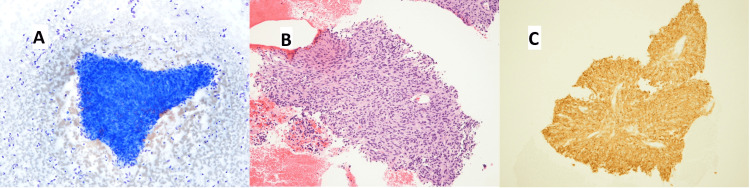
Fine-needle aspiration biopsy of the hepatic lesion A) Diff-quick smear showed a spindle cell cluster. B) Routine hematoxylin and eosin (H&E) cell block showed spindle cells. C) Immunostain for CD117 (c-Kit) was diffusely positive.

A percutaneous fine needle aspiration (FNA) biopsy was performed on a hepatic lesion. This revealed spindle cells diffusely positive for cluster of differentiation 117 (CD117 or c-Kit) consistent with a diagnosis of GIST (Figure 2).

**Figure 3:**
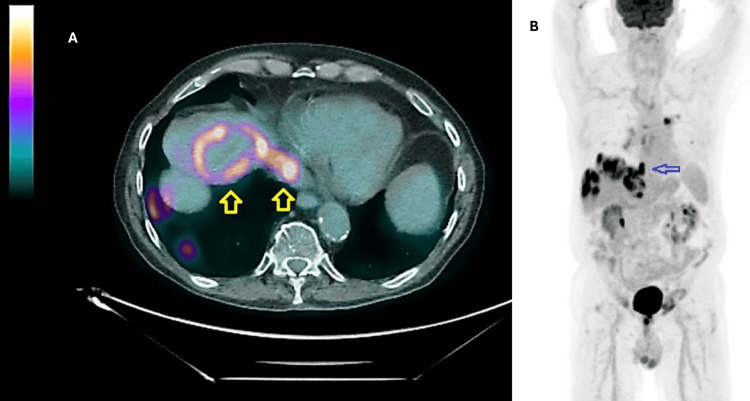
Positron-emission tomography (PET) scans demonstrating intravascular tumor extension A) A fused, axial PET CT scan of the abdomen showing intense 18-F fluorodeoxy glucose (FDG) uptake along a hepatic tumor lesion and extending along the middle hepatic vein into the inferior vena cava (arrows). B) A coronal PET scan demonstrated an FDG-intense band extending from liver lesion into the inferior vena cava toward the right side of the heart (arrow).

Next generation sequencing of the tumor sample revealed c-Kit mutations: duplication in exon nine of six nucleotides at c.1504_1509 resulting in p.A502_Y503 duplication and a missense mutation of the exon 17 at c.2459 resulting in an aspartate to glycine switch. The contiguous band-like extension of the tumor invading the IVC was also demonstrated on a positron-emission tomography (PET) scan (Figure 3A, 3B).

He was subsequently started on ripretinib. 

**Figure 4:**
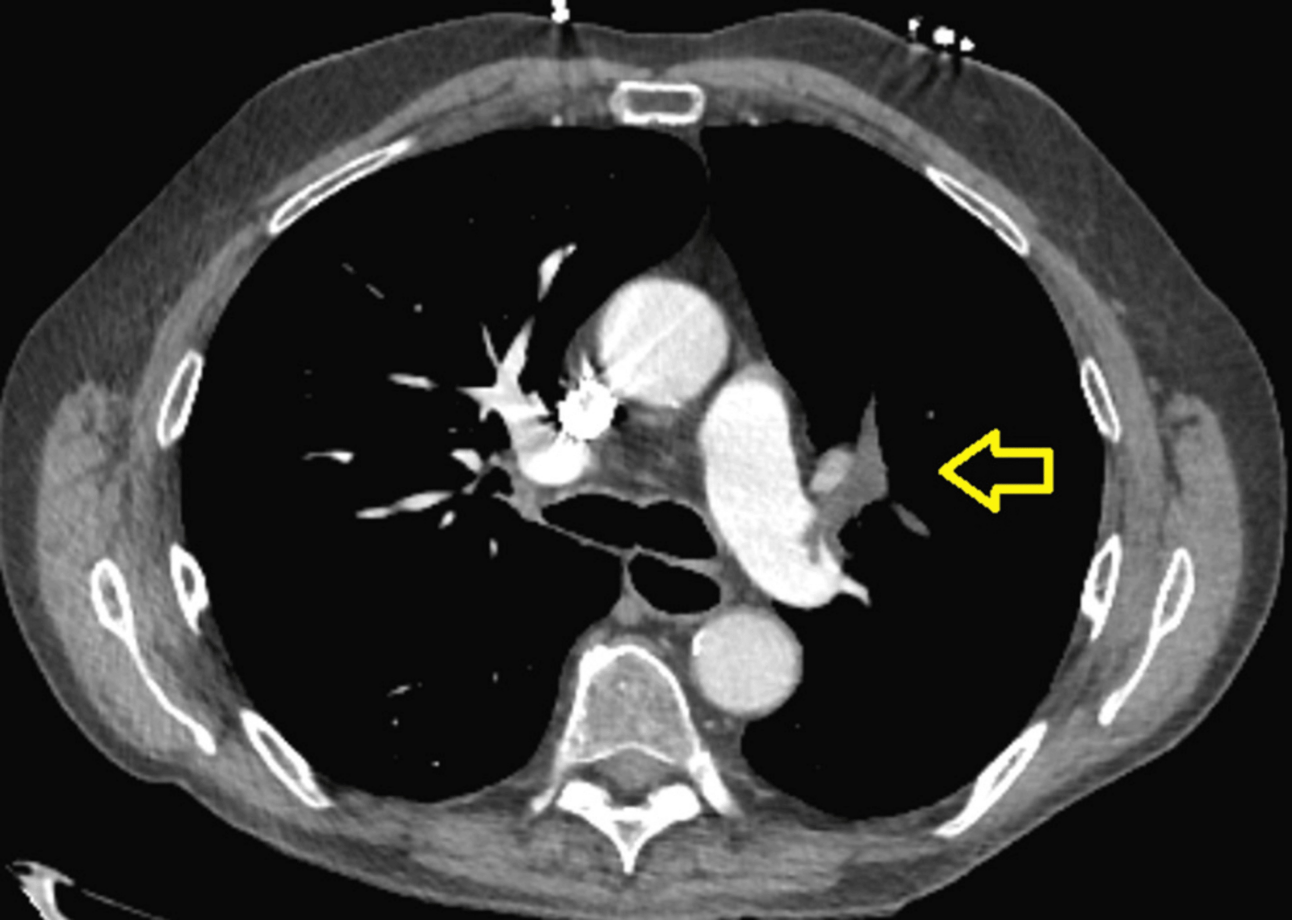
A CT angiogram of the chest The scan revealing a moderate-sized acute pulmonary embolus involving the left upper lobe branches of the pulmonary arterial system measuring 3 cm in length and 0.8 cm in diameter. There were smaller and more peripheral emboli within the sub-segmental branches in the upper lobes bilaterally.

On arrival, he was afebrile, tachycardic with a heart rate in the 110s, and requiring supplemental oxygen via nasal cannula at four liters (two liters at baseline). The CT pulmonary angiogram revealed an embolus measuring 3 cm x 0.8 cm involving the pulmonary artery branches of the left upper lobe with smaller peripheral emboli in the sub-segmental branches of the same lobe (Figure 4).

A right heart strain was ruled out by a normal serum troponin, B-type natriuretic peptide, and normal transthoracic echocardiogram. The patient was started on a heparin drip and admitted to the intensive care unit for closer monitoring. Mechanical thrombectomy was not pursued as he was hemodynamically stable. A venous Doppler scan of his lower extremities was negative. He had been on apixaban prior to admission, and was switched to therapeutic subcutaneous low-molecular weight heparin (enoxaparin) on discharge. Of note, this was his fourth presentation for pulmonary embolism in two years with no documented evidence of deep vein thrombosis in any of his extremities. 

**Figure 5:**
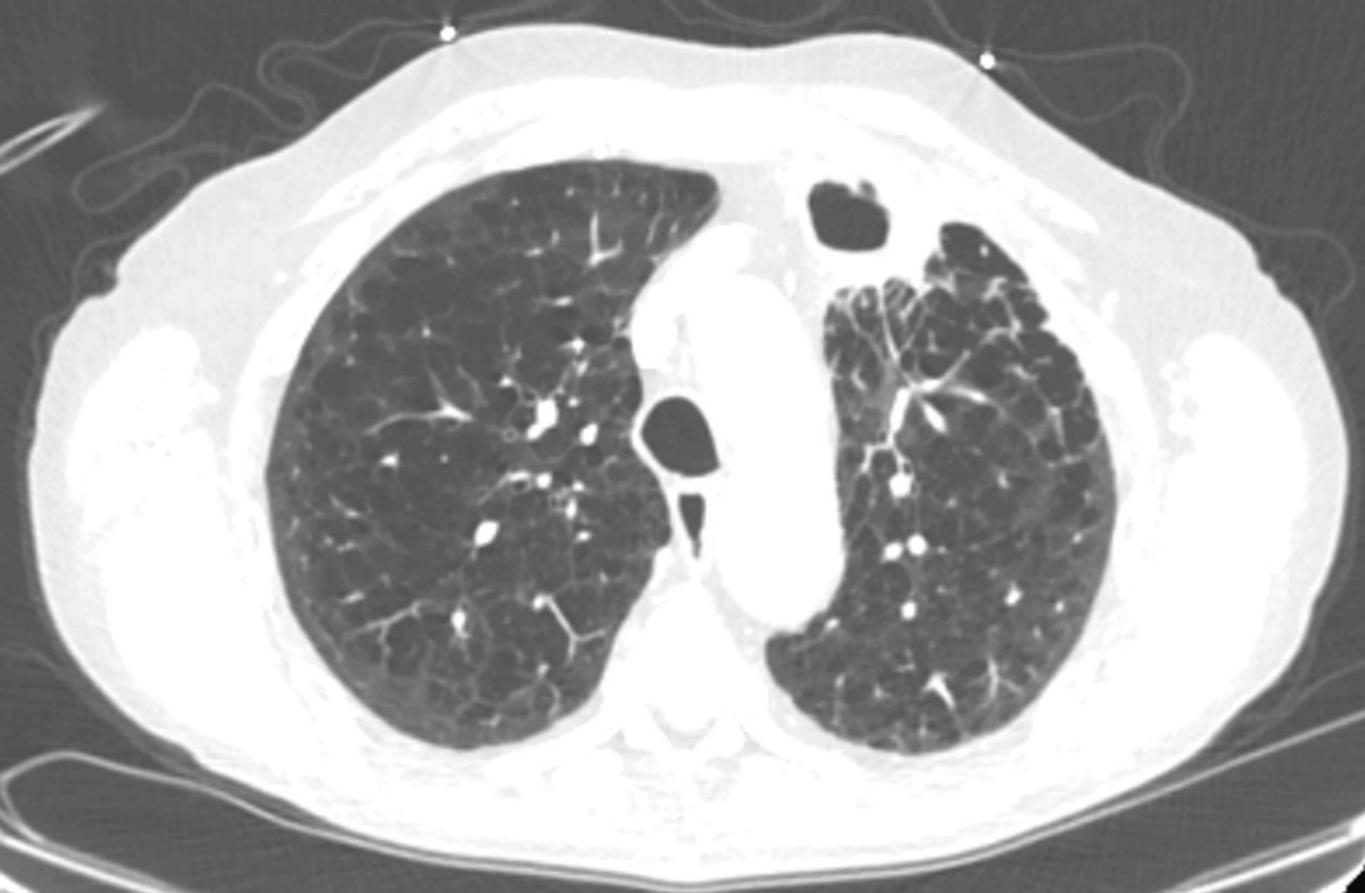
A contrast-enhanced CT scan of the chest The scan showing a cavitary lesion in the anterior segment of the left upper lobe measuring 3.8 cm x 4 cm in the antero-posterior by transverse dimensions, respectively.

Two months later, he presented with pleuritic chest pain. A CT of his chest revealed a new cavitary lesion in the anterior segment of his left upper lobe, in the same vascular territory as his embolic occlusion (Figure 5).

A workup of the cavity, including a bronchoscopy with an endobronchial ultrasound-guided FNA revealed acute inflammation and necrosis and was negative for infection. The cavity self-resolved over the next two to three months, however the branch artery to the anterior segment of the left upper lobe remained occluded on subsequent scans. These findings were suggestive of a bland pulmonary infarction.

**Figure 6:**
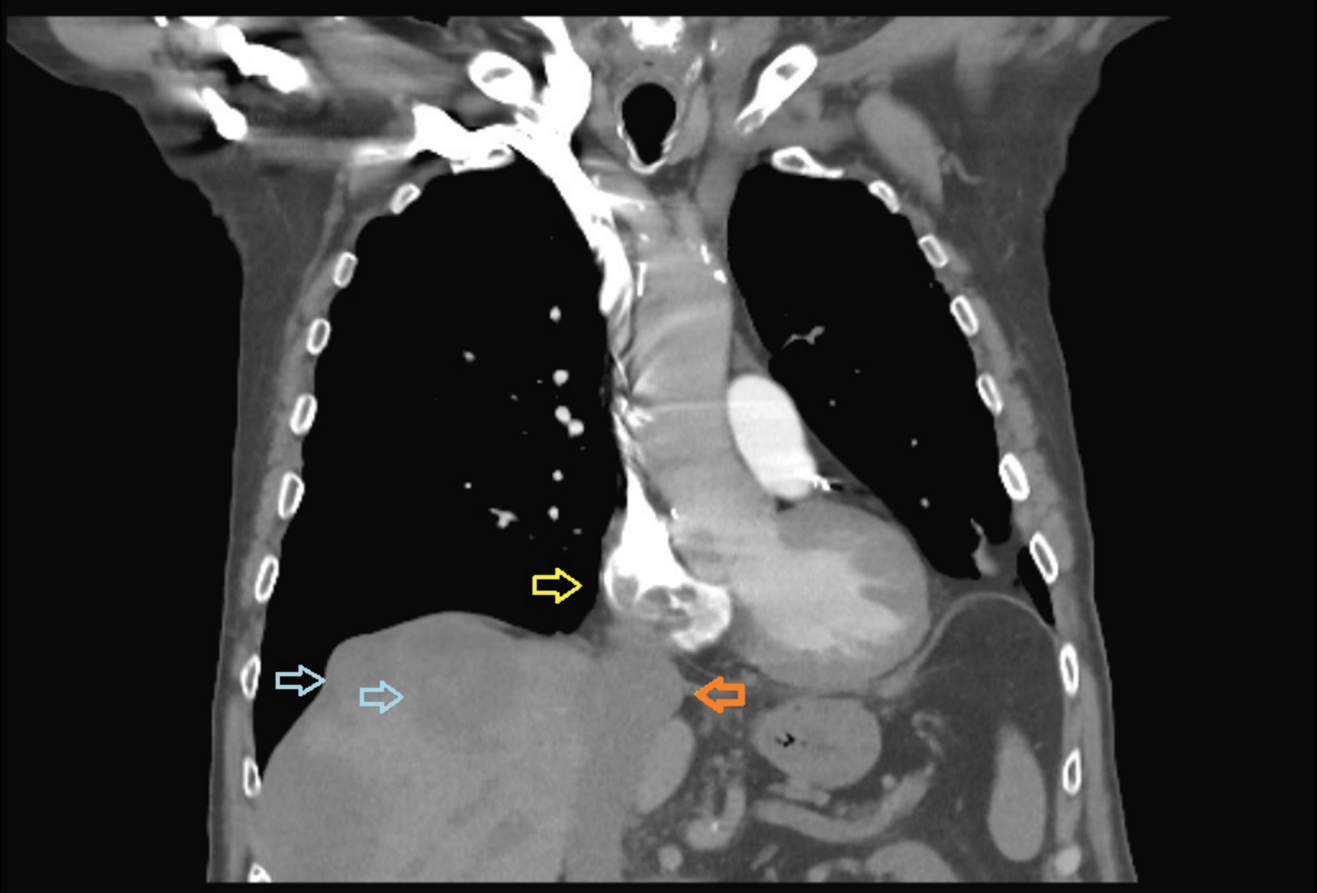
CT chest with contrast, coronal section CT scan showing the right atrial extension of the tumor thrombus. The yellow arrow indicates filling defects in the right atrium due to the tumor thrombus. The blue arrows indicate hypoattenuating hepatic lesions. The orange arrow indicates the distended inferior vena cava (IVC) with the tumor thrombus.

During this period, the tumor thrombus in his IVC continued to grow. He was switched from ripretinib to regorafenib, but failed to respond. He was admitted to the hospital two months later with new bilateral pulmonary emboli despite being adherent to subcutaneous enoxaparin. A CT scan from this presentation revealed that the tumor thrombus was now extending to the right atrium (Figure 6).

The patient had a prolonged hospital stay and eventually elected for hospice care. He passed away shortly after in the hospital.

## Discussion

Here we present a rare example of a tumoral vascular invasion by GIST and putative tumor embolism in an antemortem case. The most common site for metastases is the liver, comprising 20-60% of cases, followed by the omentum and mesentery [4-6]. Rare sites of metastases include the bone and the lung [6]. As per the Tumor, Grade, and Metastasis (TGM) staging proposed by Woodall et al., metastatic disease of any size and grade is considered stage IV and independently associated with inferior survival [7]. A Surveillance, Epidemiology, and End Results (SEER)-based study showed that the median overall survival in patients with GIST and hepatic metastases was 49 months [8]. It is interesting to note that the individual in the current study lived for about 15 years after the diagnosis of hepatic metastases. An invasion of the IVC by GIST is extremely rare with only four cases reported previously and two of these examples have been included in the references [9,10]. VTE attributed to GIST is also very rare, with only six prior cases [11]. Mutations of exon 9 have been associated with a poor prognosis compared with mutations in exon 11 and tend to exhibit resistance to imatinib. This appears to be true in our case too [12,13].

The gold standard for diagnosis of tumor embolism is histopathologic demonstration of the tumor in the pulmonary vasculature [1,14]. Autopsy case series have shown that tumor embolism is highly underdiagnosed and that a low threshold for suspicion is required due to its similarity to thromboembolism [3,14]. Although there was no direct histopathological evidence for IVC invasion and later right atrial invasion in the current case, the radiological features were highly suggestive given the contiguous nature of the mass with the hepatic lesions and the shared F-fluorodeoxyglucose (FDG) avidity on the PET scan. This, combined with recurrent episodes of pulmonary embolism on anticoagulation and pulmonary infarction all point toward episodes of tumor embolism with or without PTTM [1,15]. Although rare, pulmonary infarction can result in cavitation [16].

The natural history of pulmonary tumor embolism is poorly understood due to a lack of antemortem diagnoses. Management is challenging, partly due to the difficulty in making a diagnosis and due to limited experience. Source control by surgical removal of the tumoral intravascular invasion or by definitive chemotherapy could prevent further episodes, but data is conflicting. Kaptein et al. performed a prospective study on 86 patients with renal cell carcinoma and tumor thrombi found at diagnosis [17]. Of these, 37 had tumor thrombi in the IVC, 34 in the renal vein, and in the remaining cases, the tumor thrombus extended above the diaphragm. Thrombectomy alone was performed in 36 cases, 15 patients received anticoagulation, and a combination was pursued in nine cases. Subsequently, these patients were observed for a median of 24 months. Nine patients developed recurrent tumor thrombi and 36 patients developed VTE. They found that having a tumor thrombus at baseline or its recurrence was associated with more VTE (adjusted HR 6.61, 95% CI 3.18-13.73). They also found that thrombectomy did not lower VTE incidence, and anticoagulation tended to lower VTE risk by half but not eliminate it. This may be attributed to the fact that on histopathology, tumor embolism is seen across a spectrum from pure tumor to predominantly thrombus [1]. They also noted that some cases labelled as VTE might, in fact, be tumor embolism. Of interest, patients with tumor thrombi on anticoagulation had a greater risk of major bleeding events compared with the total cohort (15.5% vs. 7.7%)[17]. This may be in part due to the high background bleeding risk in patients with renal cell carcinoma. A pediatric case series suggested that in cases where the tumor thrombus is located below the level of the renal veins, IVC filter placement can be a protective bridging strategy while a definitive treatment of the tumor is pursued [18].

## Conclusions

To conclude, we present a rare case of a metastatic GIST invading the IVC associated with recurrent pulmonary embolism. Pulmonary tumor embolism is clinically very similar to VTE and is highly under-recognized. Certain features of this patient's presentation are highly suggestive of pulmonary tumor embolism but do not rule out VTE. These features include a) imaging evidence of intravascular tumor invasion initially into the IVC and then into the right atrium; b) a history of multiple episodes of embolism while on anticoagulation; c) lack of evidence for deep vein thrombosis of the extremities; and d) chronic pulmonary vascular occlusion and associated pulmonary infarction and cavitation.

Management options include a reduction of the tumor burden by surgery or chemotherapy depending on disease stage. Anticoagulation is partially effective in preventing further episodes. IVC filter placement can be considered for temporary prevention of embolism in cases where the tumor invasion is limited to below the renal veins.
